# Automatic classification of protein structures relying on similarities between alignments

**DOI:** 10.1186/1471-2105-13-233

**Published:** 2012-09-14

**Authors:** Guillaume Santini, Henry Soldano, Joël Pothier

**Affiliations:** 1, Université Paris 13, Sorbonne Paris Cité, Laboratoire d’Informatique de Paris-Nord (LIPN), CNRS(, UMR 7030), Villetaneuse, F-93430, France; 2 UPMC, Université Paris 06, Atelier de BioInformatique, F-75005 Paris, France

## Abstract

**Background:**

Identification of protein structural cores requires isolation of sets of proteins all sharing a same subset of structural motifs. In the context of an ever growing number of available 3D protein structures, standard and automatic clustering algorithms require adaptations so as to allow for efficient identification of such sets of proteins.

**Results:**

When considering a pair of 3D structures, they are stated as similar or not according to the local similarities of their matching substructures in a structural alignment. This binary relation can be represented in a graph of similarities where a node represents a 3D protein structure and an edge states that two 3D protein structures are similar. Therefore, classifying proteins into structural families can be viewed as a graph clustering task. Unfortunately, because such a graph encodes only pairwise similarity information, clustering algorithms may include in the same cluster a subset of 3D structures that do not share a common substructure. In order to overcome this drawback we first define a *ternary similarity* on a triple of 3D structures as a constraint to be satisfied by the graph of similarities. Such a ternary constraint takes into account similarities between pairwise alignments, so as to ensure that the three involved protein structures do have some common substructure. We propose hereunder a modification algorithm that eliminates edges from the original graph of similarities and gives a reduced graph in which no ternary constraints are violated. Our approach is then first to build a graph of similarities, then to reduce the graph according to the modification algorithm, and finally to apply to the reduced graph a standard graph clustering algorithm. Such method was used for classifying ASTRAL-40 non-redundant protein domains, identifying significant pairwise similarities with Yakusa, a program devised for rapid 3D structure alignments.

**Conclusions:**

We show that filtering similarities prior to standard graph based clustering process by applying ternary similarity constraints i) improves the separation of proteins of different classes and consequently ii) improves the classification quality of standard graph based clustering algorithms according to the reference classification SCOP.

## Background

During the past decade the databases of protein sequences have grown exponentially reaching several millions entries while 3D protein structures databases grew quadratically so as to reach, regarding the Protein Data Bank (PDB) [1],∼30000 non redundant structures sharing less than 90% sequence identity. In order to assign a structure and then a function to as many new sequences as possible, there are various methods. When a sequence is similar enough to the sequence of one or more known 3D structures, methods based on homology modeling give satisfying results. When sequence similarity fall in the “twilight zone” - *i.e.* under 30% of sequence identity - one has to resort to other methods. Among those, threading methods take advantage of available 3D structures to infer a 3D structure from a new sequence. Using statistical filters parametrized on a library of structural cores -*i.e.* a bank of invariant structural motifs of protein families -, they correlate 1D (i.e. sequential) and 3D information. In such context, the predictive ability of the threading method directly depends on the representativeness and exhaustivity of the core library. Such a library can be built upon a set of representative structures taken from expert structural classifications [2,3] as SCOP [3] and CATH [4]. However, due to the necessary careful manual inspection of the data, these expert classifications face difficulties in coping with the growing number of newly determined protein structures. For instance, since the last version of SCOP (1.75), there has been a growth of about 21% (10417 to 12643) of the total number of non-redundant protein chain in the PDB ( VAST [5] non-redundant set for a BLAST p-value of 1^0−7^available at ftp://ftp.ncbi.nih.gov/mmdb/nrtable/). Hence automatic and fast clustering approaches become necessary.

Over the past decade there have been many attempts aiming at developing automatic classification procedures, mainly applying supervised classification methods using as labels of know 3D structures part of a reference classification. Jain and Hirst [6] proposed such a supervised machine learning (ML) algorithm based on random forest to learn how to classify a new 3D structure in a SCOP family. Thus a 3D structure is described using a set of global structural descriptors composed from four to six secondary structural elements (SSEs) for protein domains. However, supervised classification methods heavily depends on the reference classification, whose labels are fixed, and therefore only partially address the problem of automatic classification of 3D structures.

Røgen and Fain [7] suggested an unsupervised approach using a description of protein structures derived from knot theory in order to describe the compared structures globally. Zemla *et al*[8] proposed a similarity scoring function that aims at automatically identifying local and global structurally conserved regions in order to drive a clustering algorithm. Sam et al. [9] investigated varieties of tree-cutting strategies and found some irreducible differences between the best possible automatic partitions and SCOP classifications. These results have been confirmed by the work of Pascual-Garcia et al. [10]. They have investigated the non-transitivity of objective structural similarity measures: a protein *A* can be found similar to an other protein *B*, the protein *B* can be found similar to a third protein _*ok*_ and still proteins *A* and *C* may share no similarity. They have shown that non transitivity, that does occur at low similarity levels, leads to non unicity of the partition resulting from the clustering process. For fine granularity -*i.e.* high similarity levels- structural transitivity is satisfied with few violations within a given cluster and different classification procedures converge to the same partition. For coarser granularities -*i.e.* lower similarity levels- as the similarity measures are computed on distorted and divergent 3D motifs, requiring to partition the set of structures implies choices for deciding which transitivity violations should be ignored. Depending on these choices classifications may differ significantly.

Furthermore, such similarity based classification procedures of 3D structures only consider a single overall pairwise similarity measure or score, that is derived from local similarities, and do not make use of the detailed mapping of similar parts computed during the alignment process. As a consequence, these procedures, ignoring the mapping information, may lead to cluster proteins that do not all share a common motif. This point will be further illustrated using a Simple case studies section. Then, prior to running a graph based clustering process, we propose to make use of the mapping information in ternary similarity constraints applied on triples of structures. Our experiments will compare the agreement between automatic classifications, obtained with and without that preliminary processing, and the SCOP reference classification.

First we need to use the similarity degree between two protein structures in order to build a graph of similarities whose vertices are protein structures and edges correspond to similarities exceeding a given threshold. Such a graph can be directly given as an input to a graph based clustering process. However, our proposal is to use the mapping information for defining similarities between protein alignment as follows. Let us define an alignment between 2 proteins *A* and *B* as a one to one mapping of (sub)parts of *A* onto (sub)parts of *B*. A similarity between two alignments is thus defined if the two alignments share a common sequence. More precisely, the alignment between protein *A* and protein *B* and the alignment between protein *B* and protein *C* are stated as similar if the (sub)parts of *B* implied in both alignments constitute a significant part of at least one of the two alignments. In other words, we consider a ternary similarity between *A*, *B* and *C*, centered on *B*, and that such a ternary similarity is stronger if the regions of *B* implied in its similarity with *A* are also implied in its similarity with *C*. The aim of the preprocessing step is then to consider that whenever there is an edge between proteins *A* and *B* and an edge between proteins *B* and *C*, then the ternary similarity centered in *B*, quantifying the common part shared by the three proteins, should be high enough. In that case we will state that the ternary constraints are satisfied. The preprocessing step will then consist in reducing the original graph to a graph satisfying the ternary constraints.

To summarize it, the method, shortly introduced in [11] starts with building a graph of 3D structures whose edges represent pairwise similarities. That graph is first transformed into its *line graph* that represents the adjacencies between the graph edges. Applying the ternary constraints results in eliminating some vertices of the line graph. A maximal line graph is then extracted from the resulting graph. The graph of 3D structures corresponding to this maximal line graph now satisfies the ternary constraints: every triple of linked proteins corresponds to a significant structural motif. In our experiments, MCL [12], a Graph Clustering algorithm previously applied with success to the clustering of protein sequences in families on a large scale [13] is used for achieving the final classification. That classification is then compared to the expert classification SCOP at the finest granularity -*ie* the SCOP “Family level”-. We also experiment a standard clustering method, suited for applications involving a large and unknown number of clusters, the preprocessing step being also applied in these experiments.

## Definitions

In this work as in [11] a protein structure is identified to an item *o*. Each item is defined as a set of parts *o*={_*pi*_}. Here each part _*pi*_ will represent a structural unit defined by a sequence of one or more amino-acids. We first define the similarity of two parts by comparing their distance to a threshold.

### Items and similarities

#### Definition 1 (Similarity of item parts)

Let _*pi*_and $p_{i}^{\prime }$ be parts of two different items, $D(p_{i},p_{i}^{\prime })$ be a distance between parts, and _*TP*_a distance threshold defined on the distance range. We define $\mathrm{simP}(p_{i},p_{i}^{\prime })$, the similarity of items parts _*pi*_and $p_{i}^{\prime }$, as follows: 

• $\mathrm{simP}(p_{i},p_{i}^{\prime })$ is *True* iff $D(p_{i},p_{i}^{\prime })\leq T_{P}$.

We also suppose that we have a mapping function *M* that maps subsets of items parts into a one-to-one correspondence. For protein sequences such a function is an alignment algorithm. Two items are then considered as similar if they have enough parts in common.

#### Definition 2 (Similarity of two items)

Let *O* be a set of items and a mapping function *M*. Let (*o*,^*o′*^) be two items, and *M*(*o*,^*o′*^) be the set of pairs of parts of *o* and ^*o′*^in one-to-one correspondence, then, the *symmetric similaritysimO*(*o*,^*o′*^) between items *o* and $o^{\prime }$ is defined as follows: 

• *simO*(*o*,^*o′*^) iff *Card*(*M*(*o*,^*o′*^))≥_*TO*_, where _*TO*_is a given threshold.

Elements of *M*(*o*,^*o′*^) are denoted as *mapped pairs*. We now define a ternary similarity relation over triples of items.

#### Definition 3 (Centered ternary similarity of items)

Let (^*o′*^,*o*,^*o′′*^) be three items such that *simO*(^*o′*^,*o*) and *simO*(*o*,^*o′′*^) are true, and _*P*^*o′*^,^*o*′^_(*o*) be the subset of parts of *o* related to both $o^{\prime }$ and ^*o′′*^, *i.e.*, such that $P_{o^{\prime },o^{\prime }}(o)=\{p\;|\;(p,p^{\prime })\in M(o,o^{\prime })$ and (*p*,^*p′′*^)∈*M*(*o*,^*o′′*^)}. Then *si*_*m*3_(^*o′*^,*o*,^*o′′*^), the *ternary similarity centered around o*, is defined as follows: 

*si*_*m*3_(^*o′*^,*o*,^*o′′*^) iff *Card*(_*P*^*o′*^,^*o*′^_(*o*))≥*T*×*min*(*Card*(*M*(*o*,^*o′*^)),*Card*(*M*(*o*,^*o′′*^))),where the ternary similarity threshold *T* lies in the range 0−1.

We note and exemplify hereunder that the notion of ternary similarity should not be confused with the notion of transitivity, which only depends on the graph of similarities, i.e. on binary relations. As an example, we consider the case of three items, pairwise linked, i.e. forming a clique, and highlight a case in which none of the three centered ternary similarities exceeds the ternary similarity threshold.

#### Property 1 (Cliques can not satisfy centered ternary similarity)

Here is a counterexample. Let (*o*={_*pi*_,_*pj*_},^*o′*^={_*pi*_,_*pk*_},^*o′′*^={_*pj*_,_*pk*_}) such that *M*(*o*,^*o′*^)=_*pi*_, *M*(^*o′*^,^*o′′*^)=_*pj*_and *M*(*o*,^*o′′*^)=_*pk*_. Assuming that _*TO*_=1 we obtain that {*o*,^*o′*^,^*o′′*^} is a 3-clique, and therefore similarity is transitive. Nevertheless *si*_*m*3_(*o*,^*o′*^,^*o′′*^) is *False*, *si*_*m*3_(^*o′*^,*o*,^*o′′*^) is *False* and *si*_*m*3_(*o*,^*o′′*^,^*o′*^) is *False* for any threshold *T*>0, and therefore all ternary constraints are violated.

### Graph model

Similarities between items are encoded as edges in an undirected graph *G* whose vertices are identified to items, and whose edges represent similarities between pairs of items. Conventional notations are those of [14].

#### Definition 4 (Graph of item similarities)

The graph *G* of item similarities with respect to the above notions of pairwise similarities on a set of *O* items is defined as follows: 

• *G*=(*O*,*E*) where *V*(*G*)=*O*and *E*(*G*)=*E*={(_*oi*_,_*oj*_)∈^*O*2^| *simO*(_*oi*_,_*oj*_) is True}.

#### Definition 5 (Independent connected components)

A connected component of *G* is a subgraph of *G* in which any pair of vertices is connected through a path. Correlatively independent connected components, named ICCs, are two subgraphs of *G* for which there is no path between any node of one component to any node of the other component.

Now we introduce a useful equivalent representation of *G* as a *line graph* whose definition is recalled here.

#### Definition 6 (The line graph of a graph)

Let *G*=(*O*,*E*) be a graph. Its line graph is defined as *L*(*G*)=(*E*,*F*) where *F*={(_*ei*__*ej*_)∈^*E*2^| _*ei*_ adjacent to _*ej*_ in *G*)}.

The line graph transformation is bijective if nodes labels are known and has the following property:

#### Property 2

The connected components of *G* and of *L*(*G*) are in a one-to-one correspondence.

Indeed, given _*gi*_and _*gj*_two ICCs of *G*, according to definition 5 there is no edge linking a node of _*gi*_ with a node of _*gj*_. Consequently, by construction, there cannot be adjacency between any edge of _*gi*_ and any edge of _*gj*_. Then, according to definition 6 there is no edge between *L*(_*gi*_) and *L*(_*gj*_). The reciprocal can easily be inferred.

Our purpose is to modify *L*(*G*) in order to satisfy the constraints derived from centered ternary similarities. Such modification relies on the following properties:

#### Property 3

Line-Graph

1. A vertex of *L*(*G*) is an edge of *G*,

2. Two connected vertices of *L*(*G*) correspond to two adjacent edges of *G*: let two edges of *G* be (^*o′*^,*o*) and (*o*,^*o′′*^), the corresponding edge of *L*(*G*) is (^*o′*^,*o*)−(*o*,^*o′′*^).

3. Removing a vertex in a line-graph *L*(*G*) leads to the line-graph of the subgraph of *G* obtained by removing the corresponding edge from *G*.

From property 3 and definition 3, the centered ternary similarity can be checked on every *L*(*G*) edge as such an edge links two vertices representing two similarities sharing a common item.

### Measures

In order to compare two classifications we use standard comparison measures of classification similarity. More precisely, let *P*={_*P*1_,_*P*2_,…,_*Pn*_} be a partition of the set of items *O*, two items _*ok*_∈_*Pi*_and _*ol*_∈_*Pj*_ are said *co-classified* iff _*Pi*_=_*Pj*_.

Let *P* be a *reference* partition and ^*P′*^be an other partition of the same set of items *O* obtained by a classification procedure. We denote as *TP* the number of pairs of items co-classified in _*Cp*_and in $C_{P^{\prime }}$, as *FN* the number of pairs of items co-classified in the reference partition *P* but not in ^*P′*^, and as *FP* the number of pairs of items co-classified in the partition ^*P′*^but not in *P*.

The *Precision* and *Recall* of the partition ^*P′*^with respect to the reference partition *P* are defined as follows: 

$\mathrm{Recal}l^{P}(P^{\prime })=\frac{\mathrm{TP}}{\mathrm{TP}+\mathrm{FN}}$,

$\mathrm{Precisio}n^{P}(P^{\prime })=\frac{\mathrm{TP}}{\mathrm{TP}+\mathrm{FP}}$.

^*RecallP*^(^*P′*^) measures the ability of the classification procedure for co-classifying item pairs when a pair is co-classified in the reference partition *P* (ability to retrieve all the positives). ^*PrecisionP*^(^*P′*^) measures the accuracy of the classification procedure to co-classify correctly item pairs according to the reference classification *P* (ability to provide a correct prediction when predicting a positive).

The Jaccard similarity coefficient [15] is defined as follows: 

$\mathrm{Jaccard}(P,P^{\prime })=\frac{\mathrm{TP}}{\mathrm{TP}+\mathrm{FN}+\mathrm{FP}}$

It is a measure of concordance between two partitions of a same set of items very similar to the F-measure. When negatives are much more numerous than positives, this measure has the advantage - over measures such as MCC (Matthews correlation coefficient) and plain accuracy - of not taking into account over-represented True Negatives. As a result, variations of concordance are easier to detect.

## Simple case studies

**Figure 1:**
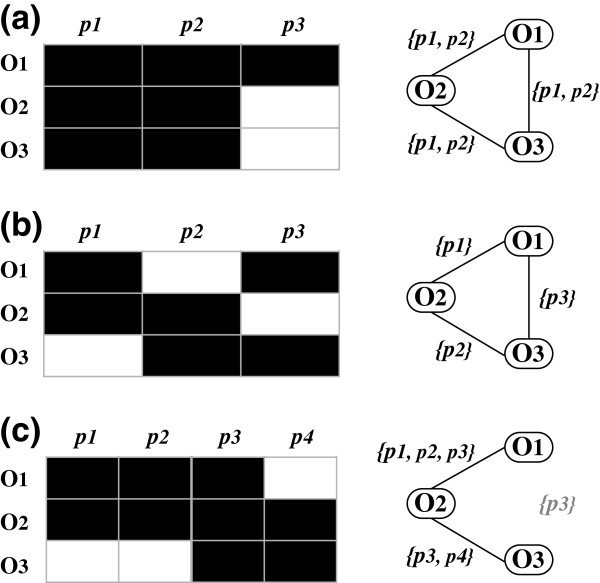
**Similarity transitivity and common sub-motif occurrence.** Description of items (_*Oi*_) and items parts (_*pi*_) parts, and corresponding graph of similarities *G*. **(a)** Transitive case with set of parts common to all items, **(b)** Transitive case with no part common to all of items; **(c)** Non-transitive case with a part common to all items.

As previously mentioned [10], similarity relations between proteins structures belonging to the same class show high values and are considered almost to be transitive, *i.e.* whenever _*o*1__*o*2__*o*3_ belongs to a given class, we should have that *simO*(_*o*1__*o*2_)∧*simO*(_*o*1__*o*3_)∧*simO*(_*o*2__*o*3_) = *True*. According to our graph formalism, these three items are represented by a 3-clique in *G* (*cf.* Figure 1-a). Clustering strategies such as search of max-cliques should allow identifying classes of proteins sharing a similar set of structural motifs, which is not the case.

For the sake of clarity the definition of items similarity for the two first case studies is simpler than definitions 1 and 2: two items are stated as similar when they share at least one identical common part.

### Case 1: Non transitive Graph *G* and no common sub parts

**Figure 2:**
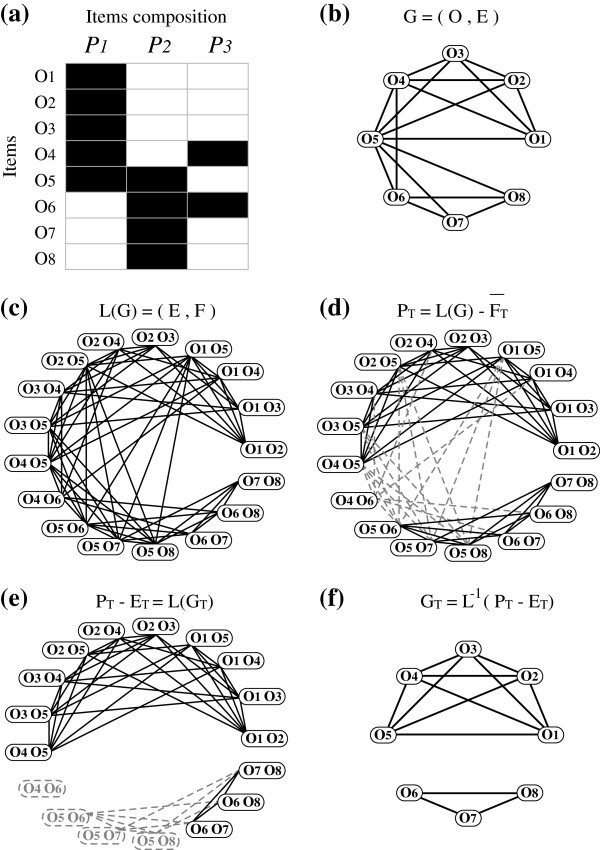
**Graph modification method.(a)** the description of object parts, **(b)** the graph *G* of pair similarities, **(c)** the line graph *L*(*G*), **(d)** the graph _*PT*_with marked edges $\bar{F_{T}}$ not fulfilling the constraint of ternary similarity represented in dashed gray, **(e)** the graph _*PT*_−_*ET*_=*L*(_*GT*_), with vertices _*ET*_removed during the heuristic **ℋ** and their removed incident edges represented in dashed gray, **(f)** the graph _*GT*_fulfilling the ternary similarity.

In Figure 2-a, considering items _*o*1_={_*p*1_}, _*o*5_={_*p*1_,_*p*2_} and _*o*8_={_*p*2_}, we have: *simO*(_*o*1_,_*o*5_) by part*s*{_*p*1_} and *simO*(_*o*5_,_*o*8_) by part*s*{_*p*2_}. An item such as _*o*5_ made of two subparts (_*o*5_={_*p*1_,_*p*2_}) is denoted as a modular item. Though _*o*5_similarities such as (_*o*1_,_*o*5_) and (_*o*5_,_*o*8_) are adjacent in *G* (Figure 2-b) they represent different local similarities: edge (_*o*1_,_*o*5_) represents part _*p*1_ and (_*o*5_,_*o*8_) represents _*p*2_. A modular item can be considered as a linker between two or more classes: it is similar, and then connected to any item member of the class 1 of items comprising part _*p*1_(*class*1=(_*o*1_,_*o*2_,_*o*3_,_*o*4_,_*o*5_)) and to any member of the class 2 of items comprising part _*p*2_(*class*2=(_*o*5_,_*o*6_,_*o*7_,_*o*8_)). Consequently its degree is higher than those of its neighbors that are only linked to members of a single class. Due to their higher degree, modular items will act as kind of “attractors” during clustering processes. Consequently immediate neighbors of different classes will tend to form around the modular item a unique class, grouping together items having nothing in common (for example _*o*1_ and _*o*8_). Thus, in such a context, direct search of the most connected components from *G* does not seem appropriate.

### Case 2: Transitive Graph *G* and no common sub parts

In Figure 1-b, considering items _*o*1_={_*p*1_,_*p*3_}, _*o*2_={_*p*1_,_*p*2_} and _*o*3_={_*p*2_,_*p*3_}, we have *simO*(_*o*1_,_*o*2_) due to part*(s)*{_*p*1_}, *simO*(_*o*2_,_*o*3_) due to part*(s)*{_*p*2_} and *simO*(_*o*1_,_*o*3_) due to part*(s)*{_*p*3_}. Here transitivity exists at the similarity graph level: _*o*1_, _*o*2_ and _*o*3_constitute a clique. Nevertheless considering similarities at the local level of shared sub parts, there is no transitivity as no sub part is shared by all of the three items, which case shows that even if transitivity is assumed at the graph level for a set of items, nothing ensures the occurrence of a set of subparts common to all items. Therefore direct search for max-cliques components from *G* does not seem appropriate.

### Case 3: Non transitive Graph *G* and common sub parts

Similarity measures used for comparing modular and fuzzy motifs must be *tolerant* to take into account the flexibility and the divergence of the compared items as in Yakusa [16], the algorithm used here for identifying, selecting and mapping similar 3D protein structures. As shown in Figure 1-c, with such a measure some similarities stated as not significant by use of user defined selection threshold may be rejected even when a sub-part is found similar. Again, for the sake of clarity the definition of items similarity in the following case study is simplified. Two items are considered as similar if at least 50% of the parts of the shortest item are mapped to sub-parts of the second item. Considering items _*o*1_={_*p*1__*p*2__*p*3_}, _*o*2_={_*p*1__*p*2__*p*3__*p*4_} and _*o*3_={_*p*3__*p*4_}, we have *simO*(_*o*1__*o*2_) and *simO*(_*o*2__*o*3_) but not *simO*(_*o*1__*o*3_), which corresponds to a non-transitive case at the graph level with the occurrence of a sub-part _*p*3_ common to all items _*o*1_, _*o*2_ and _*o*3_. In such a case, the search for max-clique is not well suited.

## Method

### Use of ternary similarities

These case studies emphasize some difficulties encountered by classical graph clustering approaches in grouping together modular items in classes where all items share a common set of parts. Searching max-clique - sets of items with transitive relations in graph *G* - does not seem adequate (*cf.* Case 2) as transitive relations in the graph may occur between items sharing no common subparts, and not be necessary (*cf.* Case 3) as items whose relations are not transitive in the graph may share a common set of sub-parts. Searching for the most connected components (*cf.* Case 1) in considering all links of the initial graph *G* is not appropriate either as some highly connected items may force the union of two significantly different classes.

These drawbacks could be corrected by searching a maximal subgraph _*GT*_ of *G* in which the ternary similarity constraint is verified, before applying any classical connectivity-based clustering approaches. Indeed, as depicted later, application of ternary similarity constraint will tend to reduce the connectivity between items not sharing a same set of subparts (Cases 1 and 2) and preserve links of interest (Case 3) increasing their relative connectivity in the context of the modified graph _*GT*_.

### Applying ternary similarity constraint

Let *L*(*G*)=(*E*,*F*) be the line graph of *G*=(*O*,*E*). From property 3 each edge of *L*(*G*)((^*o′*^,*o*),(*o*,^*o′′*^)) links two similarities having one item in common and can be submitted to the ternary similarity test. The edges of *L*(*G*) are then divided into the subset _*FT*_of *F* whose elements satisfy the ternary constraints and the subset $\bar{F_{T}}$ whose elements will be marked: 

• _*FT*_={((^*o′*^,*o*),(*o*,^*o′′*^))∈*F* | *si*_*m*3_(^*o′*^,*o*,^*o′′*^) is True}

• $\bar{F_{T}}\cup F_{T}=F$,

The *graph of pairs*_*PT*_is obtained by deleting marked edges from *L*(*G*): 

•_*PT*_=(*E*,_*FT*_), *i.e.*$P_{T}=L(G)-\bar{F_{T}}$.

The modified graph _*PT*_is no more homomorphic to a line graph, *i.e.* there is usually no graph ^*G′*^ such that _*PT*_=*L*(^*G′*^). The bijection established by the line graph transformation between *L*(*G*) and *G* is broken by the introduction of the ternary similarity constraints. We will search now for a maximal line graph *L*(_*GT*_) that is a subgraph of _*PT*_. As the edges of *L*(_*GT*_) are also edges of _*PT*_, the ternary relations for the corresponding items (^*o′*^,*o*,^*o′′*^) will necessarily hold in _*GT*_. For that purpose a greedy heuristic **ℋ** eliminates vertices of *L*(*G*) until it finds a subgraph, with no marked edges, corresponding to a line graph *L*(_*GT*_) of some subgraph _*GT*_ of *G* (*cf.* property 3.3).

### Heuristic for selecting a subgraph of *L*(*G*) homomorphic to a line graph with no marked edges

Let _*NT*_ be the marked subgraph of *L*(*G*), *i.e.*_*NT*_=*L*(*G*)−_*PT*_ and $E(N_{T})=\bar{F_{T}}$. Let us recall that *L*(*G*)−^*E′*^ where ^*E′*^⊆*E* is the subgraph of *L*(*G*) induced by ^*E′*^(*L*(*G*)−^*E′*^ contains all edges of *L*(*G*) that join two vertices in ^*E′*^). We will search for some - minimal - subset _*ET*_of _*NT*_ vertices such that *L*(*G*)−_*ET*_contains no marked edges, and therefore, following property 3.3, corresponds to the line-graph of some - maximal - subgraph _*GT*_ of *G*.

Removing first the vertices of _*NT*_showing the maximal degree maximizes the ratio of the number of deleted vertices over the number of edges taking away the graph from a line graph. As minimizing _*ET*_is equivalent to maximizing *L*(_*GT*_) it is also equivalent to maximizing _*GT*_. This subgraph of *G* both fulfills the ternary similarity constraint and tends to be maximal. 

 1/ *N*←_*NT*_*//initializes N as the set of marked edges of L(G) //*

 2/ _*ET*_←*∅// initializes the set of vertices to be removed //*

 3/ **while***E*(*N*)≠*∅*: *// still some marked edges //*

*// identification of*_*NT*_*vertices of maximal degree//*

 4/ *Δ*(*N*)← the maximal degree among *N* vertices,

 5/ _*Ed*_←{*e* | *e*∈_*ET*_and *deg*(*e*)=*Δ*(*N*)}

 6/ *N*←*N*−_*Ed*_

 7/ _*ET*_←_*ET*_∪_*Ed*_*// iterative definition of*_*ET*_*vertices set//*

## Material

SCOP database is an expert classification of structures of protein domains. It is used as a source of data for our clustering studies and as reference classification to which classes formed by clustering procedure are compared to.

SCOP offers a hierarchical classification organized as a 6-levels tree. Protein domains are successively divided into “Classes”, “Folds”, “SuperFamilies” and “Families”. The leaves of the tree are the protein domains. In this study automated classifications will be compared to the finest grained SCOP level, a group of protein domains belonging to the same SCOP Family are then considered as a SCOP cluster.

The set of items is taken from 3D protein structure of domains of SCOP database [3]. Over the 488.567 available domain structures we restrict our search to a non-redundant subset made of the 10.569 SCOP domain representatives exhibiting less than 40% sequence identity - *i.e.* the ASTRAL_40 data set (version 1.75) [17].

The mapping function of two objects is performed by the YAKUSA software [16]. The program searches for the longest common similar substructures, between the query structure and every structure in the structural database. Such common substructures consist of amino-acids of proteins *o* and ^*o′*^ and are represented by the mapped parts *M*(*o*^*o′*^).

The set of protein pairs showing a YAKUSA z-score over or equal to _*TO*_=7.0 defines the edges *E* of our graph *G*.

**Figure 3:**
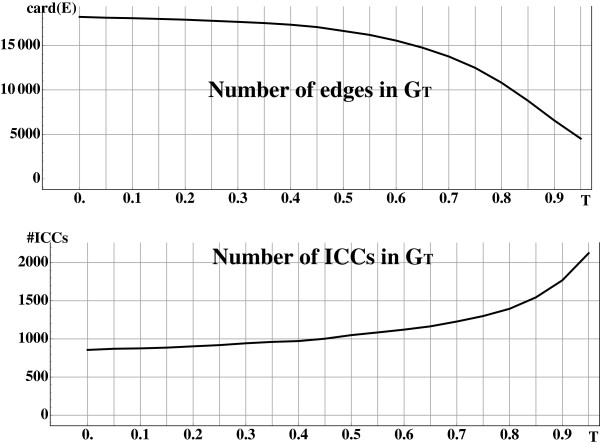
**Modification of the graph*G*.** Evolution of the size (top) and the number (bottom) of independent connex components (ICCs) of the modified graph _*GT*_for increasing threshold of ternary similarity *T*. (top) Higher is the threshold more stringent is the constraint and higher is the number of deleted edges from original graph of pair similarities *G*. (bottom) As a direct consequence, graph _*GT*_becomes more and more sparse, and connected components more numerous.

**Figure 4:**
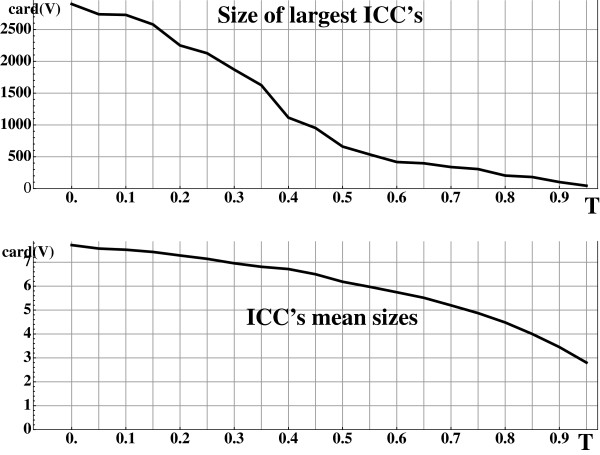
**Sizes of the connected components of the modified graph _*GT*_.** (top) Size of the largest independent connex components (ICCs) and (bottom) mean size of independent connex components of the modified graph _*GT*_for increasing threshold of ternary similarity *T*. The sparsification of the graph _*GT*_for more stringent threshold reduce both the size of the largest ICCs and the mean sizes of the ICCs.

Before applying the graph modification method we remove all the isolated proteins (proteins not similar to any other protein of the database), *i.e.* we remove all objects *o* such that *deg*(*o*) = 0. We obtain then the graph *G*(*O*,*E*) representing the pairwise similarities between 6606 items (proteins) encoded in 18199 edges (*cf.* Figure 3). Items are grouped into 856 connected components with a large component containing 2901 items (*cf.* Figure 4), achieving a initial coarse grained clustering.

## Results

### Clustering effect of the modification graph process

In order to experiment the method, *G* was submitted to the modification process using different values of the ternary similarity threshold *T* ranging from *T*=0.05 to *T*=0.95 by step of 0.05.

The heuristic **ℋ** selecting vertices _*ET*_ to be removed from _*PT*_ can potentially select any vertex (_*oi*_,_*oj*_). If (_*oi*_,_*oj*_) is the only vertex where item _*oi*_ appears, deletion of (_*oi*_,_*oj*_) leads to removal of item _*oi*_. As _*GT*_is built from the inverse line-graph transformation (every vertex of _*PT*_−_*ET*_ leads to an edge of _*GT*_), item _*oi*_ is absent from _*GT*_vertices.

By construction, our modification graph process implies a reduction of *G* connectivity. This results from removal of marked edges ( $P_{T}=L(G)-\bar{F_{T}})$ and then of vertices of _*PT*_ that kept the graph away from a line graph (*L*(_*GT*_)=_*PT*_−_*ET*_). Removal of vertices from _*PT*_ corresponds to the removing of edges from *G* to _*GT*_. As expected, this loss of connectivity is directly correlated to the value of threshold *T*. Higher values of *T* lead to a more stringent constraint of ternary similarity, and finally to a less connected graph (*cf.* Figure 3-top).

Moreover, ICC’s formed in the building of _*PT*_are transferred to *L*(_*GT*_) and from property 2 to _*GT*_. As shown in Figure 3-bottom and 4 this leads to a pre-partition of the objects. More stringent constraint of ternary similarity leads to more ICC’s of lower sizes facilitating the work of the clustering algorithm.

### Pre-clustering effect of ternary similarity constraints

Our modification graph process implies two edge deletion steps. First step is the suppression of *L*(*G*) edges failing at the centered ternary similarity test. Second step is the removal of *L*(*G*) nodes through application of the heuristic **ℋ**. According to property 3, node removal from *L*(*G*) is equivalent to edge removal from *G*.

**Figure 5:**
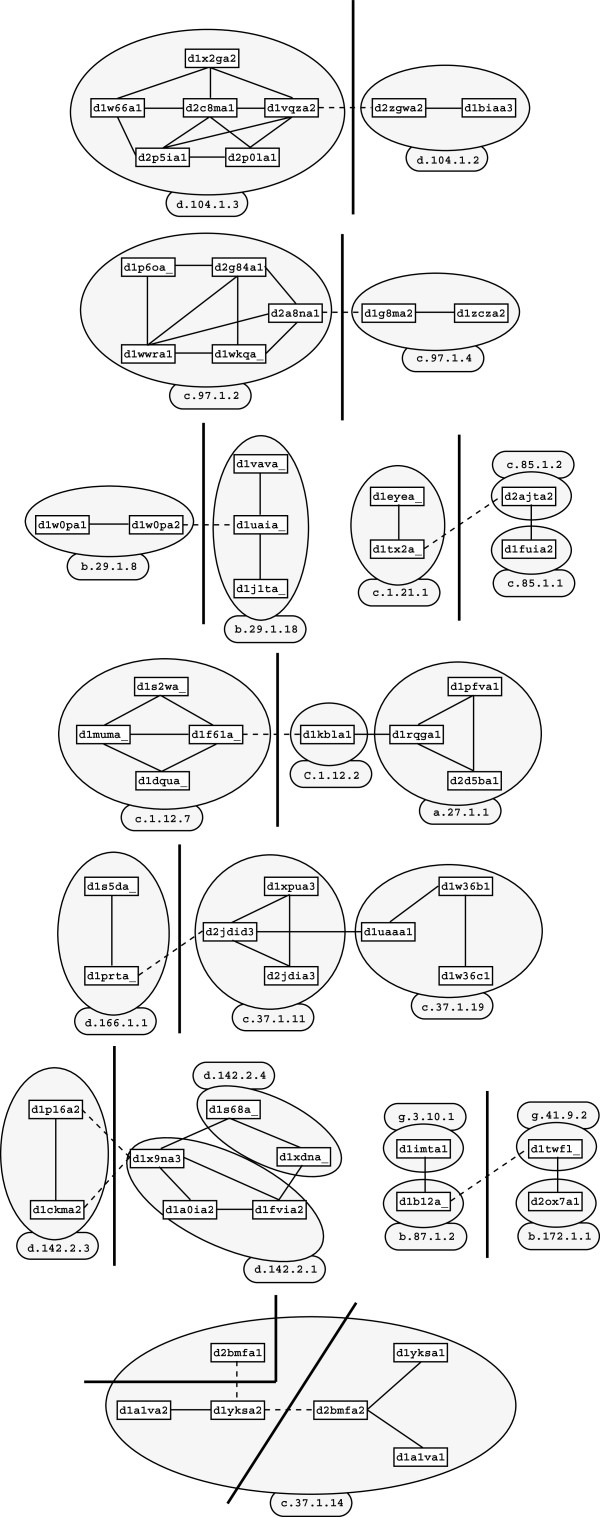
**Connected components split during graph modification ***G→***_***G*0.65**_.** Cuts of the ICC’s are represented by the thick (vertical) lines. Links removed (resp. kept) by the modification are shown in dashed (resp. continuous) lines. Items belonging to the same SCOP Family are circled in gray and SCOP Family is given in caption.

In the second step, edge deletion can potentially split an ICC of *G* into one or more ICC’s in _*GT*_. For a similarity threshold of *T*=0.65, nine ICC’s are split into two or three ICC’s. As shown in Figure 5, in eight cases, the deleted edge isolates a group of items of the same SCOP Family from items classified differently, showing that application of ternary similarity constraint tends to separate items that are to be found in different SCOP Families.

One can notice in this Figure that protein domains from different SCOP Classes are linked in *G*. This is due to the flexibility of the YAKUSA similarity measure. Hopefully, the ternary constraint identify some of these issues, and do remove such links.

### Ternary similarity threshold and 3D structural comparisons

**Figure 6:**
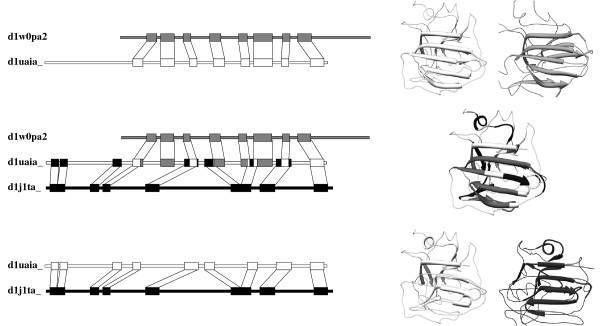
**Protein domain structures comparison in the ternary relation context.** Domains d1w0pa2 (Sialidase with SCOP “Family” Id. b.29.1.***8***), d1uaia_ (Polyguluronate lyase with SCOP “Family” Id. b.29.1.***18***) and d1j1ta_ (Alginate lyase with SCOP “Family” Id. b.29.1.***8***) are respectively figured in gray, white and black. (Top) 73 amino acids long mapped parts of (d1uaia_, d1w0pa2) pairwise alignment, (Bottom) 75 amino acids long mapped parts of (d1uaia_, d1j1ta_) pairwise alignment, and (Middle) 35 amino acids long mapped parts of d1uaia_ common to both pairwise alignments. (Left) mapped parts are represented by boxes along the domain sequence and no-mapped parts are represented by a line. (Right) mapped parts are represented by a stylized ribbon according to its secondary structure, and non-mapped parts are represented with a thin licorice. For the ternary point of view (middle), only the domain common to the two pairwise alignments is represented (d1uaia). In the 3D structures, the blocks mapped in the two alignments are shown in white, the blocks mapped only in the top alignment (d1uaia_, d1w0pa2) are shown in gray and the blocks mapped only in the bottom alignment (d1uaia_, d1j1ta_) are shown in black.

Picked-up from one of the nine splits presented in Figure 5, Figure 6 illustrates the way the fractional ternary similarity threshold identifies the candidate edges to be deleted in the context of the ternary relation. Considering the three domains d1w0pa2, d1uaia_ and d1j1ta_, pairwise similarities are significant: 73 amino acids are mapped in the alignment (d1w0pa2,d1uaia_) and 75 amino acids are mapped in the alignment (d1uaia_,d1j1ta_). But considering the ternary relation, one considers the overlap of mapped part on the common domain d1uaia_, and finds only 48% (35 aa) of the amino acids common to both alignments. Therefore, with a threshold T=0.65=65%, the ternary similarity is considered to be not significant (48*%*≤*T*) and one of the two edges of the ternary relation (d1w0pa2,d1uaia_,d1j1ta_) has to be deleted. There, the heuristic selects the edge (d1w0pa2,d1uaia_) splitting the iccs into two components according to SCOP classification.

### Classifications granularities

Application of ternary similarity constraints has a clustering effect taking into account shared similarities. It bears an incidence on the classes formed by MCL, the main clustering algorithm of our procedure. Granularity of the clustering has been studied for varying thresholds of ternary similarity T and inflation parameter *I* (*cf.* Figure 4).

The inflation parameter *I* is the main MCL parameter that rules the clustering granularity. Lower values of *I* lead to coarser clustering. Different values of *I* were tested (*I*∈[1.2,2.0] by step of 0.1 and *I*∈[2.0,3.0] by step of 0.2).

**Figure 7:**
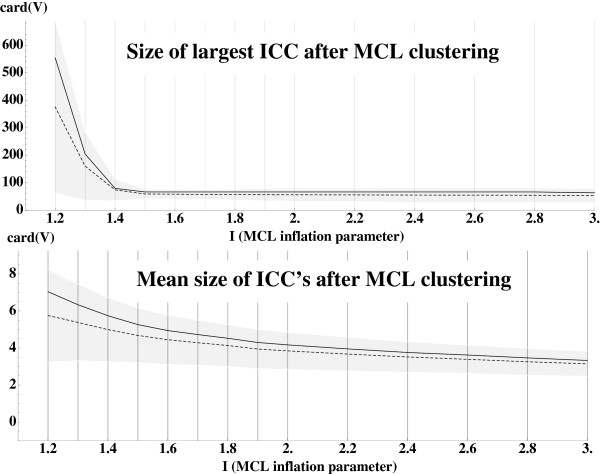
**MCL classifications.** Mean sizes (dashed lines) and ±2*σ*- standard deviation - (gray zones) of the greatest (top) and mean (bottom) MCL cluster sizes, obtained from the subgraph _*GT*_, when the ternary similarity threshold *T* varies in the range 0.05-0.95, as a function of the inflation parameter *I*. Greatest and mean MCL cluster sizes obtained from *G* are also reported (continuous lines).

As expected, large ICC’s are rapidly split into small clusters when inflation parameter increases as shown in Figure 7-top. The size of the largest clusters formed for low inflation parameters 1.2<*I*≤1.4 (coarsest granularity) depends directly on the ternary similarity threshold used which rules the granularity of the pre-clustering process. For higher inflation parameters (fine granularity) the sizes of the largest clusters appear to be almost independent from *T*, and the cluster mean size (Figure 7-bottom) is also independent from *T*.

Thus, if the reduction of *G* to _*GT*_changes the clustering of items, the granularity is not significantly affected.

### Comparison of the MCL classes to standard expert classifications

We compare the MCL classifications obtained with or without the application of ternary similarity constraints to the reference classification SCOP. This is done by mean of Precision/Recall (PR) curves rather than by ROC curves because i) the information contained in both curves are quite equivalent [18] and ii) PR curves are usually preferred in a context where the number of negative examples greatly exceeds the number of positives examples, which is the case here.

**Figure 8:**
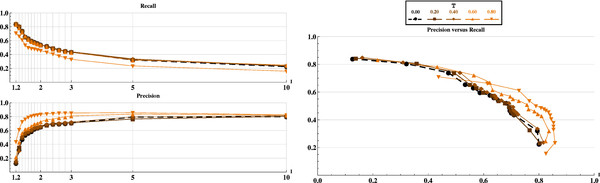
**Comparison of MCL classes to reference classification SCOP.** Comparison of classes obtained from application of MCL to the initial graph *G* (black dashed line) and modified graph _*GT*_(continuous lines). Lighter continuous lines correspond to more stringent ternary similarity constraint (varying from 0.2 to 0.8 by step of 0.2). Left top (resp. bottom): represents Recall (resp. Precision) for increasing values of parameter I (MCL inflation parameter). Right: Precision versus Recall curves points, from right to left, correspond to decreasing inflation parameter I.

As shown in Figure 8-left, increasing values of MCL inflation parameter *I*-*i.e.* making smaller clusters-, in- crease (*cf.* Precision) the ability to provide a correct prediction when co-classifying two items , and decreases (*cf.* Recall) the ability to retrieve all the positives. As ex- pected, the recall decreases when the precision increases.

Differently, for increasing values of threshold *T* (triplet must share higher similarities), precision increases, but surprisingly, this gain in precision is not correlated to a loss of recall. Indeed, for *T* in range 0.0-0.6, the recall remains stable up to high values of *T*=0.8 (corresponding to very high required similarities between triplets alignments). As a consequence, ternary constraints allow increasing the precision while preserving the recall. As shown in Figure 8-right, we can consider the use of ternary similarity as an improvement of the classification (PR curves are shifted toward the upper-right part of the graphic when using increasing values of *T*).

### Choice of the final clustering algorithm

In order to evaluate the real impact of the ternary similarity constraint independently from the choice of the final clustering algorithm, we compared classifications obtained with MCL to those obtained with a standard approach. We used a normalized spectral clustering algorithm [19] with a final k-means clustering initialized with centroids [20] computed from a hierarchical clustering of our data [21].

**Figure 9:**
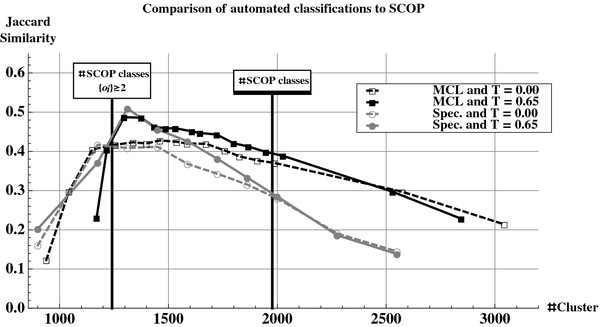
**Impact of final clustering algorithm.** Jaccard similarity coefficient between reference classification SCOP and MCL (in black) or spectral clustering (in gray) automated classifications obtained with no ternary similarity constraint (dashed lines) or with a ternary similarity constraint T=0.65 (continuous lines). The vertical line at 1977 clusters (resp. 1241 clusters) gives the number of classes in SCOP (resp. with more than one protein domain).

Both MCL and Spectral methods do not tend to form clusters with only one member. As shown in Figure 9, for a number of clusters between 1100 and 1450 - close to the number of clusters found in SCOP at the “Family level” and having more than one member (1241) - MCL and Spectral Clustering algorithms give very similar results, applying or not the ternary constraint. For a number of clusters closest to the real number of represented SCOP Families (1977), MCL algorithm gives better results and appears to be more robust.

Whatever the final clustering algorithm, Figure 9 highlights the enhancement of the quality of the automated classification procedure (with respect to SCOP reference) introduced by the ternary similarity constraint.

## Discussion and conclusions

Classification of objects such as protein structures based on pairwise similarity relations is a classical problem. We have shown the advantages of applying ternary similarity constraints in the clustering process.

The method proposed here is in line with many *constrained clustering* methods as recently investigated [22]. However in most of these methods, only *pairwise constraints* are considered: a must-link (_*ML*_) constraint states that two objects should be placed in the same cluster while a cannot-link (_*CL*_) constraint states that two objects should not be placed in the same cluster. Constraints acting on groups of objects have also been considered, as *ε*-constraints and *δ*-constraints. However both can be represented as conjunction or disjunction of pairwise constraints. Indeed it should be clear that the method proposed here deals with *ternary constraints* that cannot be represented as any combination of pairwise constraints. Besides the ternary constraints introduced here concern the initial graph representation of data: they are not constraints for which satisfaction is required (or maximized) in the clustering result. As a matter of fact, the initial graph representation, by directly linking only nodes that are similar enough, exerts some pairwise constraints on clustering: obviously two nodes belonging to two different connected components are submitted to a _*CL*_constraint. This is true for any graph based clustering approach. In such approaches, the similarity (or distance) matrix defines the initial weighted graph, and edges are then removed until the graph is partitioned. For instance in [23,24] a minimum spanning tree (in term of distances) is computed, and then using some similarity threshold, a forest is obtained. However, for large datasets, starting from a sparse graph by first applying some simple neighborhood criteria, as we do here, is a much more efficient procedure (see for instance [25] about clustering results dependency on such *sparsification* preprocessing). It would be interesting to investigate the use of our ternary constraints on various graph-based clustering schemes, as long as objects are modular. In biology, beyond protein structures, adding ternary constraints would also be relevant for clustering protein sequences using graph based methods [26].

## Competing interests

The authors declare that they have no competing interests.

## Author’s contributions

J P, HS and GS conceived the graph based algorithm. GS implemented the algorithm and carried the experiments. All authors read and approved the final manuscript.
